# Mutation Frequency of the Major Frontotemporal Dementia Genes, *MAPT*, *GRN* and *C9ORF72* in a Turkish Cohort of Dementia Patients

**DOI:** 10.1371/journal.pone.0162592

**Published:** 2016-09-15

**Authors:** Gamze Guven, Ebba Lohmann, Jose Bras, J. Raphael Gibbs, Hakan Gurvit, Basar Bilgic, Hasmet Hanagasi, Patrizia Rizzu, Peter Heutink, Murat Emre, Nihan Erginel-Unaltuna, Walter Just, John Hardy, Andrew Singleton, Rita Guerreiro

**Affiliations:** 1 Department of Genetics, Institute for Experimental Medicine, Istanbul University, Istanbul, Turkey; 2 Behavioural Neurology and Movement Disorders Unit, Department of Neurology, Istanbul Faculty of Medicine, Istanbul University, Istanbul, Turkey; 3 Department of Neurodegenerative Diseases, Hertie Institute for Clinical Brain Research, University of Tübingen, Tübingen, Germany; 4 DZNE, German Center for Neurodegenerative Diseases, Tübingen, Germany; 5 Department of Molecular Neuroscience, Institute of Neurology, University College London, London, United Kingdom; 6 Department of Medical Sciences and Institute of Biomedicine – iBiMED, University of Aveiro, 3810–193 Aveiro, Portugal; 7 Laboratory of Neurogenetics, National Institute on Aging, National Institutes of Health, Bethesda, Maryland, United States of America; 8 Institute of Human Genetics, University of Ulm, Ulm, Germany; Institut Pasteur de Lille, FRANCE

## Abstract

*‘Microtubule-associated protein tau’ (MAPT)*, *‘granulin’ (GRN)* and ‘*chromosome 9 open reading frame72’* (*C9ORF72)* gene mutations are the major known genetic causes of frontotemporal dementia (FTD). Recent studies suggest that mutations in these genes may also be associated with other forms of dementia. Therefore we investigated whether *MAPT*, *GRN* and *C9ORF72* gene mutations are major contributors to dementia in a random, unselected Turkish cohort of dementia patients. A combination of whole-exome sequencing, Sanger sequencing and fragment analysis/Southern blot was performed in order to identify pathogenic mutations and novel variants in these genes as well as other FTD-related genes such as the ‘*charged multivesicular body protein 2B’ (CHMP2B)*, the ‘*FUS RNA binding protein’ (FUS)*, the ‘*TAR DNA binding protein’ (TARDBP)*, the ‘*sequestosome1’ (SQSTM1)*, and the ‘*valosin containing protein’ (VCP)*. We determined one pathogenic *MAPT* mutation (c.1906C>T, p.P636L) and one novel missense variant (c.38A>G, p.D13G). In *GRN* we identified a probably pathogenic TGAG deletion in the splice donor site of exon 6. Three patients were found to carry the GGGGCC expansions in the non-coding region of the *C9ORF72* gene. In summary, a complete screening for mutations in *MAPT*, *GRN* and *C9ORF72* genes revealed a frequency of 5.4% of pathogenic mutations in a random cohort of 93 Turkish index patients with dementia.

## Introduction

Frontotemporal dementia (FTD) is associated with frontal and temporal lobe degeneration resulting in progressive personality/behaviour changes, and impairment of language functions [1]. FTD can be pathologically described based on the accumulation of abnormal protein as tau positive or tau-negative/ubiquitin-positive inclusions (comprising TAR DNA-binding protein 43 and FUS inclusions). The most common genetic causes of FTD are mutations in ‘*microtubule-associated protein tau’ (MAPT)*, *‘granulin’ (GRN)*, and hexanucleotide repeat expansions in *‘chromosome 9 open reading frame72’ (C9ORF72)*. These mutations account for 20–30% of the familial and 5–10% of sporadic FTD cases [2].

The *MAPT* gene is localized on chromosome 17q21 and consists of 16 exons [3]. In the human brain, tau has six different isoforms that are generated by alternative splicing of exons 2, 3, and 10. In addition to these isoforms, splicing of exons 4a, 6 and 8 also produce different *MAPT* transcripts not being expressed in the central nervous system. Although *MAPT* mutations, which account for 2–11% of all FTD cases [4], are mainly found in individuals with typical FTD, mutations in individuals with progressive supranuclear palsy (PSP), corticobasal degeneration (CBD), mild late-onset parkinsonism, and dementia with epilepsy, have also been identified [5]. Most mutations are located in exons 9–13 encoding the microtubule binding domains (that mediate interaction of Tau with microtubules) and flanking regions. The vast majority of the mutations include missense, deletion or silent mutations or mutations located close to the splice donor site of intron 10. These mutations show their effect through a toxic gain of function mechanism, either by reducing the ability of Tau to interact with microtubules or by affecting exon 10 splicing [6].

The *GRN* gene is located on chromosome 17q21 and consists of 13 exons of which the first exon and part of exons 2 and 13 are noncoding. Progranulin (PGRN) protein is involved in development, wound repair, inflammation and tumour genesis [7]. In the central nervous system, PGRN is expressed in the cerebral cortex, the hippocampus and the cerebellum; hence reduced levels of PGRN could affect both neuronal survival and central nervous system inflammatory processes [8]. The clinical spectrum of FTD associated with *GRN* mutations includes the behavioural variant (bvFTD), primary progressive aphasia (PPA), and dementia associated with movement disorders such as parkinsonism including corticobasal syndrome. The frequency of *GRN* mutations in FTD populations varies between 5–10% [9, 10].

GGGGCC hexanucleotide expansions in the first intron of the *C9ORF72* gene have been recently shown to be the most common genetic abnormality in FTD and amyotrophic lateral sclerosis (ALS). Repeat expansions were observed in 7–11% of all FTD and 12–25% of familial cases [11]. Prevalence differences can be seen among distinct geographical regions and there is a significant clinical heterogeneity within families [11]. The clinical phenotype associated with these expansions is mostly characterized by FTD symptoms and signs of motor neuron disease. The clinical presentation may be initially diagnosed as Alzheimer’s disease (AD), mild cognitive impairment (MCI), or dementia with Lewy bodies (DLB) [12]. The minimal size of a GGGGCC pathogenic repeat is under debate: some studies consider repeats of >30 GGGGCC hexanucleotide repeat units as pathogenic, whereas others use a cut-off of 60 GGGGCC hexanucleotide repeat units [13]. Currently, the detailed pathobiological mechanisms of the *C9ORF72* gene repeat expansion in neurodegeneration is not totally understood.

In early stages, AD and FTD may share clinical features, at times making it difficult to differentiate between the two diseases. Several recent studies reported mutations in *MAPT*, *GRN* and *C9ORF72* associated with clinically diagnosed AD patients [2,13]. Cruchaga and colleagues suggested in their study that in late-onset AD, mutations in *MAPT* and *GRN* may be as common as mutations in ‘*amyloid beta precursor protein’* (*APP)*, *‘presenilin 1’ (PSEN1)*, and *‘presenilin 2’ (PSEN2)*, the classical gene mutations associated with familial AD [14].

In order to determine the frequency of genetic mutations in the *MAPT*, *GRN* and *C9ORF72* genes in the Turkish dementia patient population, we used a combination of different sequencing and expansion analyses techniques in a cohort of 95 dementia patients from 93 families, clinically diagnosed mostly with AD (n = 54, 56.8%) or FTD (n = 28, 29.5%). Additionally, we also searched for mutations in other, much less frequent FTD-related genes such as the ‘*charged multivesicular body protein 2B’ (CHMP2B)*, the ‘*FUS RNA binding protein’ (FUS)*, the ‘*TAR DNA binding protein’ (TARDBP)*, the ‘*sequestosome1’ (SQSTM1)*, and the ‘*valosin containing protein’ (VCP)*.

## Materials and Methods

The study was approved by the Ethics Committee of Istanbul Faculty of Medicine, Istanbul University. A neurologist took the necessary clinical information after obtaining informed written consent from the patients and their participating family members. Consent was provided by the Legally Authorized Representative for subjects unable to consent.

Our initial cohort of dementia patients (n = 105) was originally assessed for definitely pathogenic classified mutations in *PSEN1 (NM_000021)* (n = 6), *PSEN2 (NM_000447)* (n = 0) and *APP (NM_001204302)* (n = 0) [15]. Further investigations revealed the c.3691C>T (p.R1231C) *‘neurogenic locus notch homolog protein 3’ (NOTCH3) (NM_000435)* mutation to cause clinically diagnosed AD in one particular family [16]. Furthermore, ‘*triggering receptor expressed on myeloid cells 2’* (*TREM2) (NM_018965)* homozygous mutations [c.97C>T (p.Q33X), c.197C>T (p.T66M), c.113A>G (p.Y38C)] were identified in three families presenting an atypical FTD phenotype [17]. The remaining 93 families comprising 95 patients where the above mutations were not found, were diagnosed clinically mostly either with AD (56.8%) or FTD (29.5%), other dementia forms (13.7%) being more rare (Table 1).

**Table 1:** Characteristics of the cohort of dementia patients analysed for mutations in *MAPT*, *GRN* and *C9ORF72*.

Number (n) of families and patients	93 families (95 patients)
Diagnoses (n of patients)	AD (n = 54; 56.8%); FTD (n = 28; 29.5%); DLB (n = 6; 6.3%); PDD (n = 2; 2.1%); CBD (n = 1; 1.1%); other dementias (n = 4, including PCA (n = 2; 2.1%), and PSP (n = 2; 2.1%))
Geographical origin (n of patients)	TR (74), TR/GR (2), TR/RO (1), TR/AF (1), TR/RUS (1), BG (4), CS (4), GR (2), BG/GR (2), RO (1), AL/MK (1), BG/RO (1), RO/UA (1)
Age at examination, average (range)	69.3 years (45–89)
Age at onset, average (range)	63.9 years (38–84)
Gender	60 females (63.2%), 35 males (36.8%)
Patients with known family history of dementia	61 (64.2%)
Patients with known parental first degree consanguinity	9 (9.4%)
Patients with suspected parental consanguinity	40 (42%)

AD: Alzheimer’s disease, FTD: frontotemporal dementia, DLB: dementia with Lewy bodies, PDD: Parkinson’s disease with dementia, CBD: cortical basal degeneration, PCA: posterior cortical atrophy, PSP: progressive supranuclear palsy, TR: Turkey, BG: Bulgaria, GR: Greece, MK: Macedonia, RO: Romania, UA: Ukraine, AF: Afghanistan, RUS: Russia, CS: Serbia and Montenegro, AL: Albania

All participants were recruited consecutively over 24 months (2010–2012) in the Behavioural Neurology and Movement Disorders Unit outpatient clinic in Istanbul Faculty of Medicine, Istanbul University and underwent detailed clinical and neuropsychological examination and, in most cases cerebral magnetic resonance imaging (cMRI) or positron emission tomography (PET) imaging. The diagnosis of dementia was based on the National Institute of Neurological and Communicative Disorders and Stroke and Alzheimer’s disease [18], FTD was defined following the criteria of International Behavioural Variant FTD Criteria Consortium [19] and the criteria developed by an international group of PPA investigators [20] and the diagnosis of DLB was made based on the consensus guidelines from the DLB consortium [21]. Diagnostic procedures for Parkinson’s disease dementia (PDD) followed the recommendations of the movement disorder society task force [22]. The study population was comprised of 60 female and 35 male patients with a mean age at onset of clinical signs of 63.9 ± 9.8 years (age ranges 38–84 years). Positive family history of dementia was reported in 64.2% (n = 61) of patients, 9.4% (n = 9) of the patients had parents known to be cousins and in 42% (n = 40) parental consanguinity of different degrees was suggested. Family history was considered as positive if at least a first or a second degree related family member was also known to suffer from dementia. To the best of our knowledge, none of the relatives of our patients was diagnosed with ALS. Most families originated from Turkey (n = 79 (85%), Table 1). Peripheral blood samples were collected and genomic deoxyribonucleic acid (DNA) was extracted by standard procedures using the Qiagen DNA isolation maxi kit (Qiagen, Hilden, Germany).

### Genetic analysis

#### Whole-exome sequencing (WES)

The DNA samples were sequenced in one flow cell lane each, on paired end 50–base pair HiSeq 2000 runs (Illumina Inc), following capture using Illumina’s TruSeq Exome Capture Kit -62Mb (Illumina, Inc) and yielding an average of about 6 billion high-quality (Phred score > = 20) bases per sample. This amount of data represented the average mean target coverage of 55x, with an average percentage of targets covered greater or equal to 10x of 59%. Sequence reads were mapped to the reference genome (human genome 19) using the Burrows-Wheeler aligner [23]. Single-nucleotide polymorphisms (SNPs) and insertions and deletions (indels) were called using the Genome Analysis Toolkit (GATK) v2.7 [24]. Visual inspection of variants was performed, when necessary, using the Integrative Genomics Viewer (IGV) v2.0 [25].

Variants were annotated using Single Nucleotide Polymorphism database (dbSNP) 138 and functional annotation was done using snpEff [26]. Variants within the genes of interest, *MAPT* (*NM_001123066*.*3)*, *GRN (NM_002087*.*2)*, *C9ORF72 (NM_018325)*, *FUS (NM_001170937)*, *TARDBP (NM_007375)*, *SQSTM1 (NM_003900*.*4)*, *VCP (NM_007126)* and *CHMP2B (NM_014043)*, were extracted for further analyses. The genes analysed presented an individual average coverage of >33x and exons in specific samples with coverage below 8x were Sanger sequenced. In order to identify potentially pathogenic variants, we assessed the frequency of the variants in the general population (Exome Aggregation Consortium (ExAC), dbSNP, 1000 Genomes Project) but they were not genotyped in a control cohort from the same population. We also evaluated the functional annotation of the variants (if the variants were synonymous, predicted to change the protein sequence or were located in either untranslated or splicing regions) and the prediction of pathogenicity (using prediction software). We considered as potentially pathogenic variants those with minor allele frequency (MAF) < = 0.1% (rare variants), predicted to change the protein sequence or to impact splicing. Mutations were considered to be definitely pathogenic if they had previously been reported in the literature as causative, or if segregation analysis confirmed pathogenicity. One new identified *MAPT* variant (c.38A>G, p.D13G) that was absent in the above mentioned populations was additionally searched in the ‘The Scientific and Technological Research Council of Turkey’ (TUBITAK)-Advanced genome and bioinformatics research centre in-house exome database of varying disorders, including neurodegenerative diseases. The data set spans 1182 individuals from Turkey who were not only genotyped for this variant, but whole exome-sequenced as part of rare disease studies. Given the high degree of consanguinity in the studied population we also used a filter for variants zygosity in order to analyse any homozygous variants of interest in these genes.

#### Sanger sequencing

We performed Sanger sequencing to confirm potentially pathogenic variants found by WES in the probands, to test family members and to sequence any exons with low coverage in WES. Exons were amplified by polymerase chain reaction (PCR; primers and conditions are available on request) using Roche FastStart PCRMaster Mix polymerase (Roche Diagnostics Corp). The PCR products were sequenced using the same forward and reverse primers with Applied Biosystems BigDye terminator version 3.1 sequencing chemistry and run on an ABI3730XL genetic analyzer as per the manufacturer’s instructions (Applied Biosystems). The sequences were analysed using Sequencher software, version 4.2 (Gene Codes).

#### Repeat-primed PCR

To screen GGGGCC hexanucleotide expansions in the *C9ORF72* gene, repeat-primed PCR was carried out as previously described [27]. Fragment length analysis was performed on an ABI 3730xl genetic analyser (Applied Biosystems, Foster City, CA, USA), and data were analyzed using Gene Mapper software. Patients were compared to a positive control sample.

#### Southern blot

A total of 10 μg of gDNA was digested with XbaI and HindIII at 37°C for 3–5 hours and electrophoresed in 0.8% agarose gel. For size determination, DNA size standards λBstEII and 2.5 kb ladder were added. After electrophoresis, the coordinates of the size standards were transferred to a stripe of white paper for the subsequent analysis of the sample results. DNA was transferred to positively charged nylon membrane. Following pre-hybridization in Church buffer at 71°C for 3 hours, hybridization was carried out at 71°C overnight. The blot was hybridized with a random primed-α^32^P-dCTP-labeled probe flanking the hexanucleotide repeat [28]. The membranes were then washed in 1% sodium dodecyl sulphate (SDS) and 0.5 M Na-Phosphate at 56–58°C. The membranes were exposed to X-ray films for 4 days at -70°C applying an intensifying screen. For measurement of the expanded alleles, the marker data (migration distance and size) from the paper stripes were transferred to semi-logarithmic graph paper. A regression curve was plotted by connecting all data points. The hybridization signals of the X-ray film were also transferred to the semi-logarithmic graph paper, pinpointing the size of the expanded (and normal) alleles.

## Results

**Fig 1:**
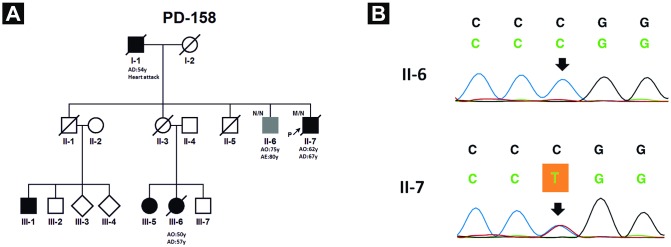
PD-158 family pedigree and sequencing chromatograms. (A) The arrow indicates the proband. Black filled symbols: patients affected with FTD; grey symbol: Alzheimer’s disease, white symbols: unaffected family members, N: normal, M: the p.P636L mutation, AO: age at disease onset, AD: age of death (B) Sequencing chromatograms of patients II-6 and II-7 showing the *MAPT* p.P636L mutation region.

In *MAPT* exon 10 we found the pathogenic c.1906C>T (p.P636L) mutation in one patient diagnosed with FTD (Fig 1). In addition, 10 variants previously reported as non-pathogenic and 1 new missense variant (p.D13G) were identified (Table 2).

**Table 2:** *MAPT* nonsynonymous variants and allele frequencies.

				All samples (n = 95)		FTD samples (n = 28)		AD samples (n = 54)		
				All (n = 95)	Familial (n = 61)	All (n = 28)	Familial (n = 20)	All (n = 54)	Familial (n = 31)	
Variant/rs ID	CADD (PHRED scaled)	In silico prediction*	Diagnosis	Counts (%)	Counts (%)	Counts (%)	Counts (%)	Counts (%)	Counts (%)	MAF**
**Pathogenic**										
c.1906C>T; p.P636L/ rs63751273	34.0	PrD/D/DC	FTD(n = 1)	1/95 (1.1)	1/61 (1.6)	1/28 (3.6)	1/20 (5)	0/54 (0)	0/31 (0)	na/na/na
**Unclear pathogenicity**										
c.38A>G; p.D13G/ new	25.3	PrD/D/DC	AD(n = 1)	1/95 (1.1)	1/61 (1.6)	0/28 (0)	0/20 (0)	1/54 (1.9)	1/31 (3.2)	na/na/na
**Non pathogenic**										
c.605C>T; p.P202L/ rs63750417	6.697	B/D/P	AD(n = 14) FTD(n = 7) PSP(n = 1) PDD(n = 1) LBD(n = 1)	24/95 (2.5)	14/61 (22.9)	7/28 (25)	6/20 (28.6)	14/54 (25.9)	5/31 (16.1)	16.5/0.2/ 0.1
c.689A>G; p.Q230R/ rs63750072	9.983	PrD/ T/P	AD(n = 6) FTD(n = 2)	8/95 (8.4)	4/61 (6.6)	2/28 (7.1)	1/20 (5)	6/54 (11.1)	3/31 (9.7)	4.2/0.04/ 0.03
c.697C>T; p.P233S/ na	na	PsD/T/P	FTD(n = 1)	1/95 (1.1)	0/61 (0)	1/28 (3.6)	0/20 (0)	0/54 (0)	0/31 (0)	na/8.835e-06/na
c.853G>A; p.D285N/ rs62063786	4.123	B/T/P	AD(n = 14) FTD(n = 8) PSP(n = 1) PDD(n = 2) LBD(n = 1)	26/95 (27.4)	17/61 (27.9)	8/28 (28.6)	7/20 (35)	14/54 (25.9)	6/31 (19.4)	16.6/0.1/ 0.1
c.866T>C; p.V289A/ rs62063787	0.001	B/T/P	AD(n = 14) FTD(n = 8) PSP(n = 1) PDD(n = 2) LBD(n = 1)	26/95 (27.4)	17/61 (27.9)	8/28 (28.6)	7/20 (35)	14/54 (25.9)	6/31 (19.4)	16.6/0.1/ 0.1
c.953C>T; p.S318L/ rs73314997	22.0	B/T/P	FTD(n = 1)	1/95 (1.1)	0/61 (0)	1/28 (3.6)	0/20 (0)	0/54 (0)	0/31 (0)	5.9/0.02/ 0.06
c.1108C>T; p.R370W/ rs17651549	33.0	B/D/P	AD(n = 4) FTD(n = 2) PSP(n = 1)	7/95 (7.4)	4/61 (6.6)	2/28 (7.1)	2/20 (10)	4/54 (7.4)	1/31 (3.2)	15.7/0.1/ 0.1
c.1321T>C; p.Y441H/ rs2258689	13.04	B/T/P	AD(n = 47) FTD(n = 25) LBD(n = 6) PDD(n = 2) PSP(n = 1) CBD(n = 1)	82/95 (86.3)	52/61 (85.2)	25/28 (89.3)	19/20 (95)	47/54 (87)	25/31 (80.6)	18.1/0.3/ 0.3
c.1339T>C; p.S447P/ rs10445337	16.56	PrD/T/P	AD(n = 16) FTD(n = 10) LBD(n = 4) PDD(n = 2) PSP(n = 1)	33/95 (34.7)	21/61 (34.4)	10/28 (35.7)	9/20 (45)	16/54 (29.6)	7/31 (22.6)	16.5/0.1/ 0.1
c.1483G>A; p.A495T/ rs63750612	0.01	B/T/P	AD(n = 1)	1/95 (1.1)	0/61 (0)	0/28 (0)	0/20 (0)	1/54 (1.9)	0/31 (0)	1.5/0.004/ 0.01

*In silico prediction programs (PolyPhen-2, SIFT, MutationTaster, respectively) were used to evaluate the effect of nonsynonymous variants on protein function and structure.

**Minor allele frequency (MAF, %) information provided from exome variant server (EVS) / ExAC / dbSNP, respectively.

Exon numbering starts with noncoding first exon EX 0. cDNA changes are relative to *MAPT* NM_001123066. Protein numbering (“p.”) refers to sequence *MAPT* NP_001116538. Abbreviations: B: benign, D: damaging, DC: disease causing, P: polymorphism, PrD: probably damaging, PsD: possibly damaging T: tolerated, na: not available.

**Fig 2:**
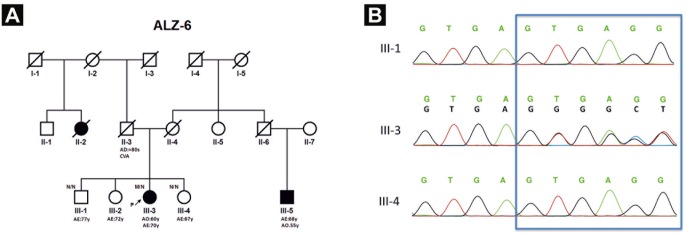
ALZ-6 family pedigree and sequencing chromatograms. (A) The arrow indicates the proband. Black filled symbols: affected patients; white symbols: unaffected family members. N: Normal, M: the c.708+6_9delTGAG deletion. CVA: cerebral vascular accident, AO: age at disease onset, AD: age of death. (B) Sequencing chromatograms show the presence of GRN c.708+6_9delTGAG deletion in the index patient III-3 that is not seen in individuals III-1 and III-4.

We identified a deletion (c.708+6_9delTGAG) in the splice donor site of *GRN* exon 6 in one patient with clinically diagnosed AD (Fig 2, S1 and S2 Figs). Alamut software (Interactive-Biosoftware, Rouen, France), which integrates different splice site prediction tools (MaxEntScan, NNSPLICE, Human Splicing Finder, SpliceSiteFinder, GeneSplicer), was used to investigate the splicing effect of this variant. These tools predicted that c.708+6_9delTGAG variant could decrease the splicing of exon 6 by 44.3%, 7.6%, 2.2%, 6.1% and 39.7%, respectively, due to decreased 5’ donor site score. Two different previously reported variants with unclear pathogenity [c.415T>C (p.C139R), c.626C>T (p.P209L)] and 6 non-pathogenic changes were found (Table 3).

**Table 3:** *GRN* nonsynonymous variants and allele frequencies.

					All samples (n = 95)		FTD samples (n = 28)		AD samples (n = 54)		
					All (n = 95)	Familial (n = 61)	All (n = 28)	Familial (n = 20)	All (n = 54)	Familial (n = 31)	
Variant/rs ID	CADD (PHRED scaled)	In silico Prediction*	Domain	Diagnosis	Counts (%)	Counts (%)	Counts (%)	Counts (%)	Counts (%)	Counts (%)	MAF**
**Pathogenic**											
c.708+6_9delTGAG/na	na	Weakens normal*** DSS	InterFB	FTD(n = 1)	1/95 (1.1)	1/61 (1.6)	1/28 (3.6)	1/20 (5)	0/54 (0)	0/31 (0)	na/na/na
**Unclear pathogenicity**											
c.415T>C; p.C139R/na	15.14	PrD/D/DC	GranF	FTD(n = 1)	1/95 (1.1)	1/61 (1.6)	1/28 (3.6)	1/20 (5)	0/54 (0)	0/31 (0)	na/0.0002/na
c.626C>T; p.P209L/ rs368995988	14.76	PrD/T/DC	GrnB	PCA(n = 1)	1/95 (1.1)	1/61 (1.6)	0/28 (0)	0/20 (0)	0/54 (0)	0/31 (0)	0.008/0.0005/ 0.0002
**Non pathogenic**											
c.99C>A; p.D33E/ rs63750742	21.4	B/T/P	ParaGran	FTD(n = 1)	1/95 (1.1)	1/61 (1.6)	1/28 (3.6)	1/20 (5)	0/54 (0)	0/31 (0)	na/6.612e-05/0.001
c.229G>A; p.V77I/ rs148531161	6.656	B/T/P	GranG	FTD(n = 1)	1/95 (1.1)	0/61 (0)	1/28 (3.6)	1/20 (5)	0/54 (0)	0/31 (0)	0.008/0.0001/ 0.0006
c.359C>A; p.S120Y/ rs63750043	9.072	B/D/P	InterGF	AD(n = 1)	1/95 (1.1)	0/61 (0)	0/28 (0)	0/20 (0)	1/54 (1.9)	0/31 (0)	0.02 /0.0008/na
c.545C>T; p.T182M/ rs63750479	8.353	B/T/P	InterFB	AD(n = 1)	1/95 (1.1)	0/61 (0)	0/28 (0)	0/20 (0)	1/54 (1.9)	0/31 (0)	0.3/0.001/ 0.002
c.1193C>T; p.S398L/ rs148213321	14.45	B/T/P	GranC	FTD(n = 1)	1/95 (1.1)	0/61 (0)	1/28 (3.6)	0/20 (0)	0/54 (0)	0/31 (0)	0.008/7.552e-05/na
c.1544G>C; p.G515A/ rs25647	5.254	B/T/P	InterDE	AD(n = 1)	1/95 (1.1)	0/61 (0)	0/28 (0)	0/20 (0)	1/54 (1.9)	0/31 (0)	1.02/0.003/ 0.005
**5’UTR variants**											
c.-203G>C/ rs555738837	14.35	na/na/na	UTR	AD(n = 2)	2/95 (2.1)	1/61 (1.6)	0/28 (0)	0/20 (0)	2/54 (3.7)	1/31 (3.2)	na/na/na
c.-22C>T/ rs572309824	12.4	na/na/P	UTR	FTD(n = 1)	1/95 (1.1)	1/61 (1.6)	1/28 (3.6)	1/20 (5)	0/54 (0)	0/31 (0)	na/na/na
c.-12C>G/na	na	na/na/P	UTR	PSP(n = 1)	1/95 (1.1)	1/61 (1.6)	0/28 (0)	0/20 (0)	0/54 (0)	0/31 (0)	na/na/na
c.-8+46G>T/ rs564341543	7.7	na/na/P	UTR	FTD(n = 2)	2/95 (2.1)	2/61 (3.3)	2/28 (7.1)	2/20 (10)	0/54 (0)	0/31 (0)	na/na/0.0002

*In silico prediction programs (PolyPhen-2, SIFT, MutationTaster, respectively) were used to evaluate the effect of nonsynonymous variants on protein function and structure.

**Minor allele frequency (MAF, %) information provided from exome variant server (EVS) / ExAC / dbSNP, respectively.

***Variant was analysed using the splicing function in Alamut software.

Exon numbering starts with noncoding first exon EX 0; Numbering according to the largest *GRN* transcript (GenBank Accession Number NM_002087.2); Numbering according to the largest *GRN* isoform (GenPept Accession Number NP_005901.2). Abbreviations: B:benign, D:damaging, DC:disease causing, P:polymorphism, PrD:probably damaging, PsD:possibly damaging T:tolerated, na: not available; DSS: donor splice site.

Three patients showed large *C9ORF72* hexanucleotide expansions (Table 4, S4 Fig). Segregation analysis could be performed in one family that had multiple family members with psychotic features (FTD-18, S5 Fig). Clinical details of family FTD-18 can be found in the S1 Table. In addition, we determined two patients with *C9ORF72* variants: c.1138T>G (p.F380V) and c.146C>G (p.T49R) (S2 Table). The former variant was identified in a Turkish patient with typical late-onset AD (74 years) who passed away in her 5^th^ year of follow up. The p.F380V variant was not found in the exome variant server (EVS), dbSNP, or ExAC databases and classified as “possibly damaging”, “damaging” and “disease causing”, by the three prediction programs PolyPhen-2, SIFT and MutationTaster respectively. The second *C9ORF72* missense variant p.T49R found in another AD patient did not segregate with the disease (the affected sister was a non-carrier) and the variant was classified as ‘benign/neutral’ by the *in silico* prediction (PolyPhen-2, SIFT). In addition, the variant is reported in the EVS (0.0154), dbSNP (0.0001731), and ExAC (0.0002) databases.

**Table 4:** Clinical information of the *C9ORF72* expansion carriers.

Patient	Gender	Origin	Age at onset (years)	Family history	Clinical diagnosis
AD-55	Female	Turkey	63	positive	FTD (PNFA)*
AD-81	Male	Macedonia	57	positive	FTD (PNFA)*
FTD-18 (II-7)	Female	Turkey	61	positive	behavioural FTD

*progressive non-fluent aphasia

We have also identified a hexanucleotide deletion in the FUS gene at the intron 5 splice donor site in a patient diagnosed with AD (S2 Table). The deletion has been reported previously and found not pathogenic based on its presence in healthy controls. In line with this, this variant was predicted to be benign according to *in silico* prediction programs, SIFT and MutationTaster.

No other pathogenic mutation in genes known to be associated with neurodegenerative diseases, and/or rare genetic causes of dementia, such as mutations *in CHMP2B*, *FUS*, *TARDBP*, *SQSTM1* and *VCP* have been identified. Furthermore, 23 variants in the *VCP* (n = 10), *C9ORF72* (n = 7), *CHMP2B* (n = 3) and *FUS* (n = 3) genes were detected, but none of them revealed as pathogenic (S2 Table).

## Discussion

In this study we performed a combination of WES and Sanger sequencing to identify pathogenic mutations and novel variants in *MAPT* and *GRN*, and fragment length analysis/Southern blot to detect pathogenic *C9ORF72* expansions in a Turkish cohort of dementia patients, the majority with a family history and shown not to carry ‘classical’ AD gene mutations. Our analysis revealed the pathogenic mutation (p.P636L) in exon 10 of *MAPT*, localized in one of the microtubule binding domains where most of the known pathogenic *MAPT* mutations are found [29]. Thep.P636L mutation has been reported in more than 30 families mainly in association with autosomal-dominant FTD, although various types of clinical presentations have been described [29]. The clinical presentation of our patient diagnosed with bvFTD is comparable to previous findings. His age at onset of clinical symptoms (62 years) and his age at death (67 years) were higher than the reported mean ages of onset and death for this mutation (52.6 and 59.3 years, respectively) [29]. The same mutation was not found in his older brother, who developed the first clinical signs much later (75 vs 62 years) and also presented a different phenotype that was compatible with a diagnosis of AD, despite the fact that vascular involvement (heart insufficiency and carotid plaques) played a significant role in his disease (Fig 1). However, additional molecular risk or co-factors responsible for the development of neurodegeneration in this family cannot be excluded.

Furthermore, we identified one new missense variant, in exon 1 (p.D13G) (Table 2): So far, the only mutations reported in exon 1 are the c.14G>A (p.R5H) [30] and the c.14G>T (p.R5L) [31], both related to a phenotype compatible with PSP. Our patient carrying the p.D13G variant had a positive family history compatible with autosomal-dominant inheritance and presented with AD. One healthy brother was found to be a non-carrier. However, the variant was not reported in the large databases and also not found in the ‘TUBITAK’-Advanced genome and bioinformatics research centre in-house exome database of varying disorders. It was classified as ‘probably damaging’ or ‘damaging’ by the prediction software (Table 2). In addition, the variant is located at a highly conserved position and might also affect the interaction of tau with tubulin and tau microtubules as it has been shown for the closely located p.R5L and p.R5H mutations [30, 31]. On this basis we suggest that the p.D13G variant is possibly pathogenic and in that case, the clinical presentation of our patient expands the clinical spectrum for mutation carriers in exon 1. However, another missense variant, located on the same amino acid (c.37G>A, p.D13N) has been reported in the ExAC database and replication and functional studies are needed to confirm our findings.

The prevalence of *MAPT*-related disorders in general is not very well known; several studies suggest that the frequency of *MAPT* mutations varies between 0 and 17.8% depending on the population [32, 33]. Taking into account FTD patients with a positive family history of dementia the frequency may increase up to 40% [33]. Our analysis revealed a mutation in the *MAPT* gene in 3.6% (1/28) of patients with FTD. This is similar to the results of previous studies performed in Sweden, US, France and US populations with reported frequencies of 0, 1.2, 2.9 and 5.9%, respectively [34, 35, 10, 31]. However, our frequency in FTD patients with positive family history of dementia was relatively low (5%, 1/20) compared to other studies with frequencies of 25, 33.3, and 40.5% respectively [36, 37, 33]. No pathogenic *MAPT* mutations were identified in our AD patients.

In the *GRN* gene we identified a deletion (c.708+6_9delTGAG) near the splice donor site of exon 6 (Fig 2, S1 Fig). This known deletion occurs in a repeat sequence (GTGAGTGA) located in the exon-intron 6 boundary. Two different studies identified similar deletions in the same repeat sequence in different positions [38, 39]. In both cases, the deletions were identified in neuropathologicaly confirmed FTD patients with TDP-43 positive inclusions. The clinical phenotype, the age at onset (60 vs mean onset: 63.5 years), and the disease evolution of our patient are similar to previously published findings [38, 39]. Both parents of our patient died at around 80 years, the father of a cerebral vascular accident and a cousin was reported to have cognitive impairment (Fig 2). Skoglund and colleagues (2011) reported that the IVS6+5_8delGTGA deletion either causes skipping of exon 6 (V200GfsX18) or retention of intron 6 (A237VfsX17) [38]. In addition, TGAG deletion carriers had modestly reduced brain mRNA levels when compared with a control patient [39]. These results are consistent with previous findings of other pathogenic *GRN* null mutations [40]. It is also noteworthy that the c.708+1G>C mutation nearby has been previously reported as pathogenic; therefore, altered splicing of exon 6 is a known pathogenic mechanism [9, 41].

We identified two additional variants in the *GRN* gene with uncertain pathogenicity (p.C139R, p.P209L). The variant p.C139R had been previously described in three families; an Italian early-onset familial FTD patient [42], a Belgian late-onset AD patient [43] and a late-onset familial FTD patient [44]. Based on protein modelling, p.C139R was predicted to be likely pathogenic because it affects folding of the PGRN by disrupting one of the cysteine disulphide bridges [36]. In addition, low plasma PGRN levels were reported in carriers of p.C139R suggesting a partial loss of PGRN function [37]. So far, though, the pathogenic nature of the mutation has not been proven by segregation analysis. We have identified this variant in a patient suffering from FTD at age of 70 years who died at age of 77 years. The patient was part of a very large family with a clear autosomal-dominant inheritance, including at least 5 more known to be affected, but already deceased family members (S3 Fig). Further analysis of additional family members failed to confirm a clear pheno- and genotype correlation in our family. However, the healthy reported variant carriers have not yet reached the age of disease onset (51, 49 and 47years) and could develop clinical symptoms.

No segregation analysis could be performed for the other unclear variant (p.P209L).

In the 5’ UTR of *GRN* we identified four variants:

The known c.-8+46G>T variant located in the promoter region is predicted to alter transcription factor binding site and has been reported in one patient with early-onset FTD (49 years) [45]. In our study this variant was observed in two families: the first patient was diagnosed with PPA and the second family was previously described by Lohmann et al. (2012) carrying the probably pathogenic *PSEN1* c.784T>G (p.L262V) mutation that segregated with the disease [15]. Based on the non-segregation of the c.-8+46G>T *GRN* variant with the disease, and the additional occurrence of the probably pathogenic *PSEN1* p.L262V mutation in the same family, we consider this *GRN* variant to be not pathogenic. Different studies suggested that variants in the 5’ regulatory region can change transcriptional activity through altered transcription factor binding sites resulting in the reduction of *GRN* expression [10, 40, 45]. They can also effect the splicing of intron 0 causing a non-functional transcript [40]. Changes in *GRN* transcriptional activity caused by variants in the promoter region might be a risk factor for the disease pathogenesis. However further gene studies are needed to show the precise effect of the promoter variants on *GRN* transcriptional activity.

The c.-22C>T variant was co-segregating with the p.C139R substitution in the family discussed above (S3 Fig). The segregation analysis of the other two variants (c.-203G>C, c.-12C>G) identified in the 5’ UTR region could not be performed due to lack of DNA from additional family members.

The frequency of 3.6% (1/28) pathogenic *GRN* mutations in our FTD cohort was very similar to previously described findings [10, 46]. Various studies showed an occurrence of *GRN* mutations between 1.3 and 11.7% [47]. Interestingly, the frequency of FTD patients with a positive family history of dementia was considerably lower compared to other studies showing an occurrence up to 25.6% [47]. No unclear or pathogenic *GRN* variants were found in patients diagnosed with AD in our cohort, except the p.P209L variant that was found in a patient with posterior cortical atrophy.

In *C9ORF72* we identified repeat expansions in 3 among 95 patients. These 3 cases presenting large expansion sizes showed clinical symptoms compatible with either behavioural or aphasic FTD (Table 4). Ages at onset ranged between 57 and 63 years and all of them had a positive family history compatible with autosomal-dominant inheritance. Co-segregation of the expansion and cognitive impairment in one of our families was explored (S1 Table, S5 Fig): By examining with revised Addenbrooke’s cognitive examination (ACE-R) the expansion-carrying children of the index patient (S5 Fig, S1 Table, II-7), we could observe a tendency to lower performance compared to non-carriers. Interestingly, many members of this family suffered from major depression, obsessive-compulsive disorder, specific and social phobia (S5 Fig, S1 Table). Psychosis and obsessive-compulsive disorder are often reported as early neuropsychiatric features in *C9ORF72*-associated bvFTD [48], but the symptoms did not segregated with the *C9ORF72* expansion in our family suggesting a different origin. To the best of our knowledge, the GGGGCC hexanucleotide repeats are the only proven pathogenic variants in *C9ORF72* reported to date and no other mutations are known. Further studies will be necessary to confirm our findings.

Our study showed that the total frequency of *C9ORF72* repeat expansions is 3.2% (3/93) in our cohort of dementia patients and 10.7% (3/28), when only taking into account patients diagnosed with FTD, but increasing to 15% (3/20) in FTD patients with a positive family history of dementia consistent with previously reported percentages [11, 49]. All together *C9ORF72* repeat expansions seem to be as frequent in Turkish FTD patients, as in other cohorts [11].

Given the high degree of families with consanguinity, we also suggested the possible presence of mutations. However at least in our cohort we could not confirm any autosomal recessive transmission due to the absence of homozygous variants classified as pathogenic. In addition, we could not find any pathogenic mutation neither in the *CHMP2B*, *FUS*, *TARDBP*, *SQSTM1* nor in the *VCP* genes, confirming their rarity also in a cohort from Turkey.

## Conclusion

This is the first molecular study in a Turkish cohort of dementia patients analysing the main FTD-related genes. *MAPT*, *GRN* and *C9ORF72* pathogenic mutations are not uncommon in our population with different types of dementia (5.4%, 5/93). When these data is combined with data we have previously published [15, 16, 17] we conclude that among 105 dementia patients, 5.7% are attributable to *PSEN1* mutations (6/105), 2.9% (3/105) to *C9ORF72* and *TREM2* each, and 0.95% (1/105) to *MAPT*, G*RN* and *NOTCH3* each, while 85.7% (90/105) remain unclear.

In this study, only taking into account patients diagnosed with FTD (n = 28), the frequency of clearly pathogenic *MAPT*, *GRN* and *C9ORF72* mutations was found to be 17.8% (5/28) showing that the frequency of both *MAPT* and *GRN* mutations is lower compared to previous findings suggesting that mutations in these genes account only for a small subset of patients in Turkey.

Looking at all results, it is apparent that 3 patients initially diagnosed with AD, including one patient with the visual variant of AD (posterior cortical atrophy), are carriers of variants in the FTD genes (*MAPT*, *GRN* and *C9ORF72*) while 5 patients diagnosed with FTD carried mutations in the AD-related *PSEN1* gene [15]. However, 3 of the patients with FTD initially received the diagnosis of AD before further disease development led to the diagnosis of FTD. Our results suggest that due to the heterogeneity and wide range of clinical presentation of both AD and FTD, routine genetic analyses should not be restricted to the well established AD or FTD genes, but use a more global approach including genes to be involved in different dementias.

Given the frequency of variants identified in *MAPT*, *GRN* and *C9ORF72*, but also in our previous analysis of *PSEN1*, *PSEN2* and *APP*, in the Turkish cohort of dementia patients studied, a standardized molecular screening procedure for these genes should be implemented in Turkey. The high birth rate expected for this and the next generation in Turkey make these studies of particularly importance given the potential high impact in genetic counseling and clinical management of FTD and dementia families.

## Supporting Information

S1 Fig Integrative genomics viewer (IGV) display of the c.708+6_9delTGAG deletion in *GRN*. The 4-bp deletion is indicated by thin black lines.
(TIFF)

S2 Fig In silico splicing effect analysis of *GRN* c.708+6_9delTGAG variant using Alamut software. The exonic regions are drawn as blue boxes. Scores from each mutation prediction tool are displayed in blue vertical bars for 5' (donor) sites, and as green vertical bars for 3' (acceptor) sites. Known constitutive signals are displayed as small blue (5') or green (3') triangles.
(TIF)

S3 Fig Pedigree of the FTD-16 Family carrying the *GRN* c.415T>C (p.C139R) and c.-22C>T variants. The arrow indicates the proband. Black filled symbols: affected patients; dark grey symbol: depressive mood; light grey symbol: mild cognitive impairment; white symbol: unaffected family members; N: wild type; M: c.415T>C (p.C139R) and c.-22C>T carrier.
(TIFF)

S4 Fig Image of Southern blot analysis. (TIF)

S5 Fig Pedigree of the FTD-18 Family carrying the *C9ORF72* repeat expansion. The arrow identifies the proband; dark grey symbol: major depression; light grey symbol: obessive-compulsive disorder; dark blue: mental retardation; M: *C9ORF72* expansion carrier; N: wild type.
(TIFF)

S1 Table Clinical phenotype of the assessed family FTD18 members. *cut-off score: 86 for age < 60 years and education > 12 years.
(DOCX)

S2 Table Variants identified in *C9ORF72*, *CHMP2B*, *FUS*, *TARDBP*, *VCP* genes and synonymous variants in *MAPT*, *GRN*. na: not available; dbSNP: single nucleotide polymorphism database; ID: identification.
(DOCX)
