# Supplementary figures and images for: Mutation Frequency of the Major Frontotemporal Dementia Genes, MAPT, GRN and C9ORF72 in a Turkish Cohort of Dementia Patients
Source: PLoS One. 2016 Sep 15;11(9):e0162592. doi: 10.1371/journal.pone.0162592 (PMC5025192; doi:10.1371/journal.pone.0162592)

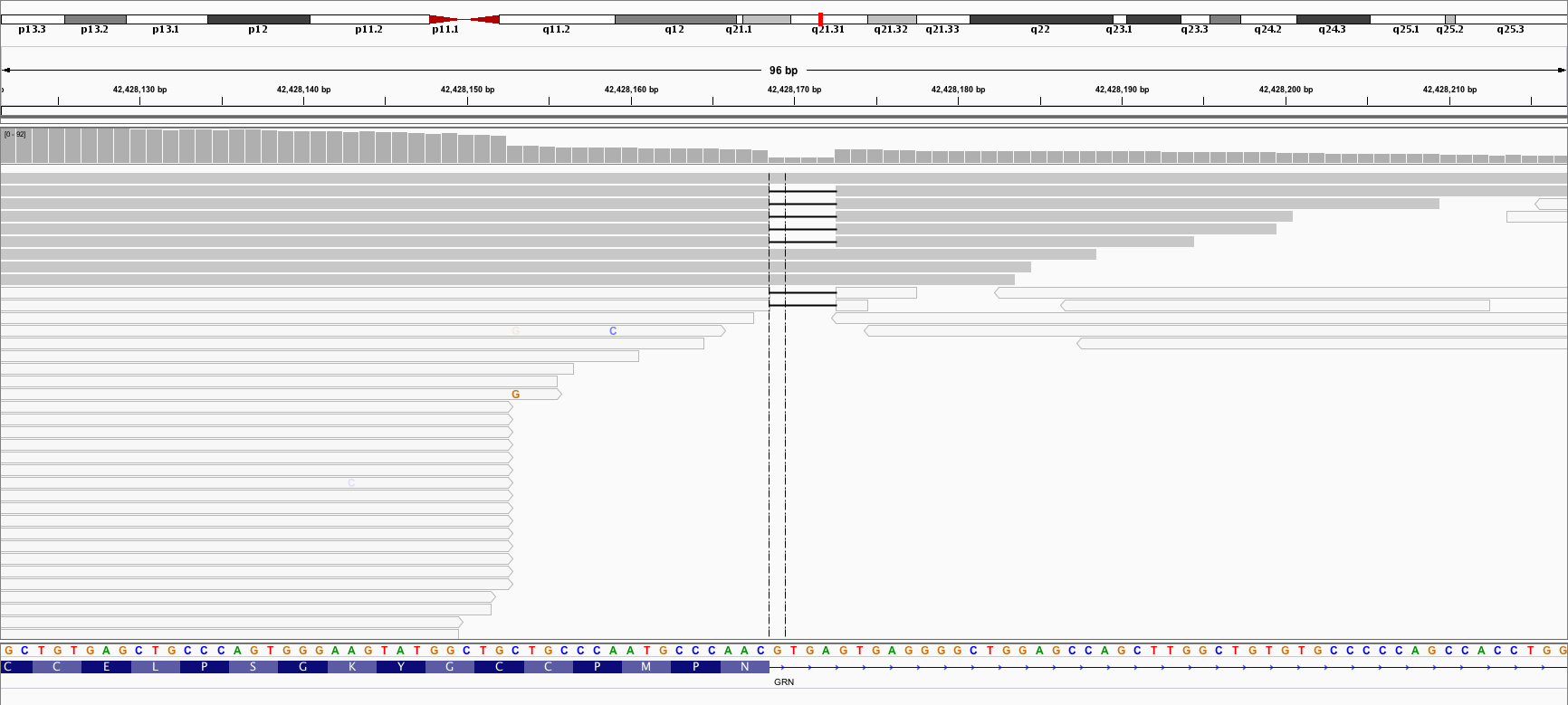

Supplement: S1 Fig — The 4-bp deletion is indicated by thin black lines. (TIFF) [file pone.0162592.s001.tiff]

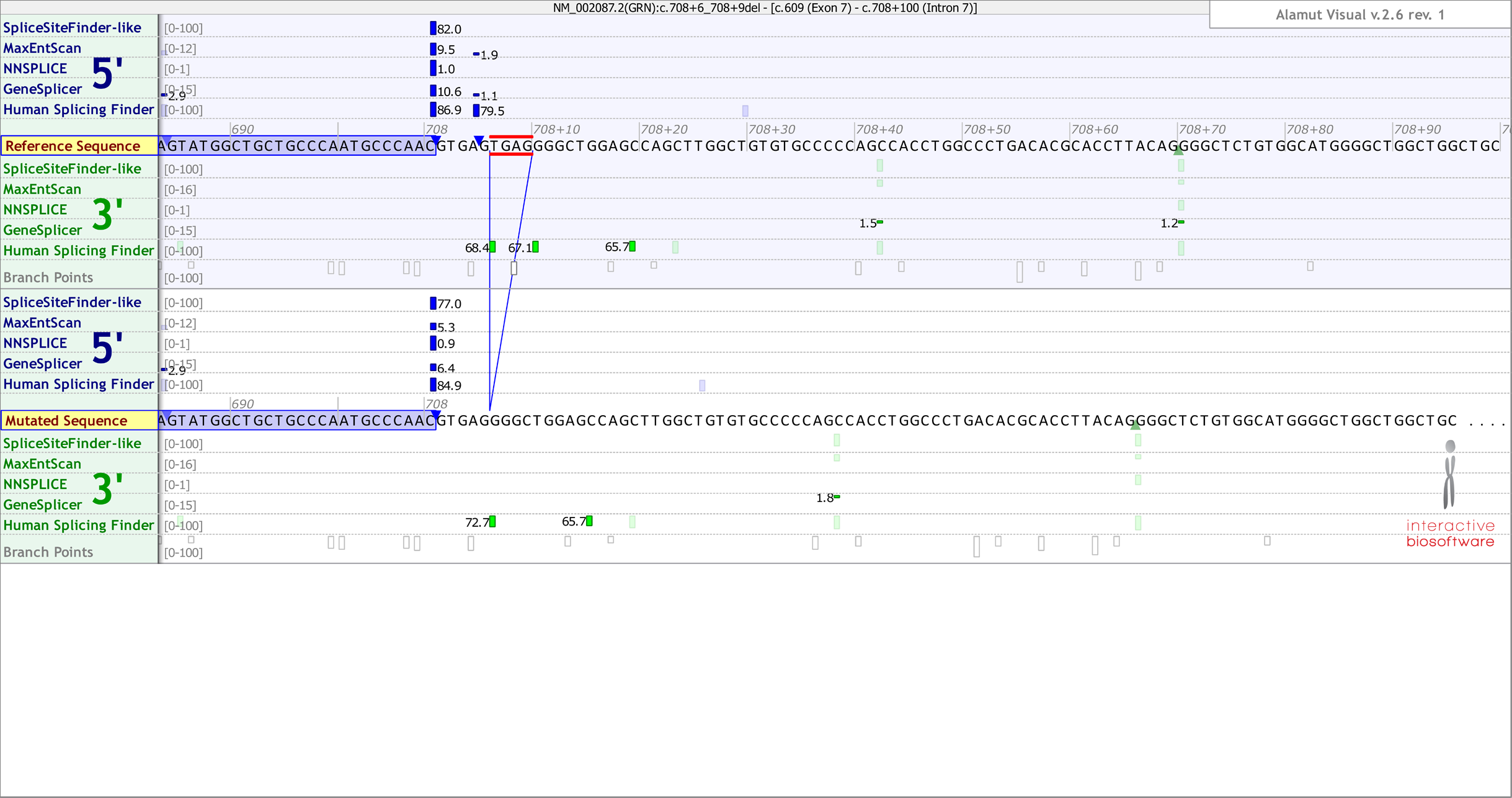

Supplement: S2 Fig — The exonic regions are drawn as blue boxes. Scores from each mutation prediction tool are displayed in blue vertical bars for 5' (donor) sites, and as green vertical bars for 3' (acceptor) sites. Known constitutive signals are displayed as small blue (5') or green (3') triangles. (TIF) [file pone.0162592.s002.tif]

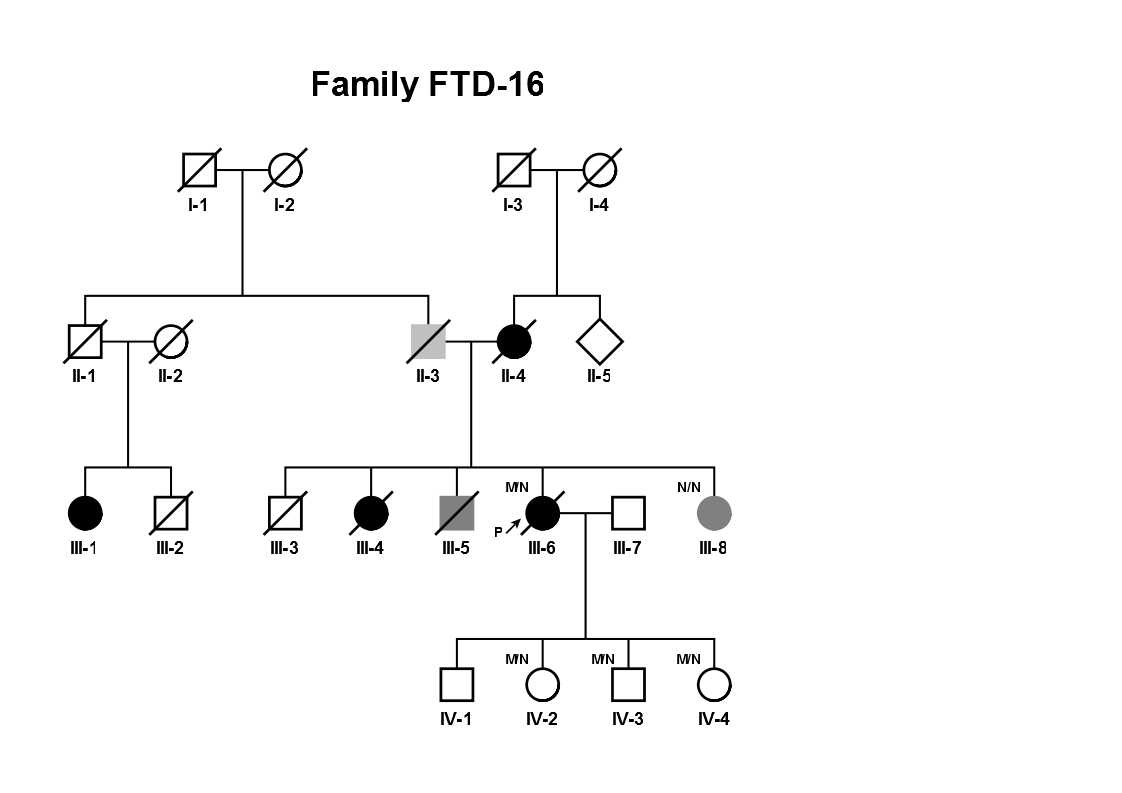

Supplement: S3 Fig — The arrow indicates the proband. Black filled symbols: affected patients; dark grey symbol: depressive mood; light grey symbol: mild cognitive impairment; white symbol: unaffected family members; N: wild type; M: c.415T>C (p.C139R) and c.-22C>T carrier. (TIFF) [file pone.0162592.s003.tiff]

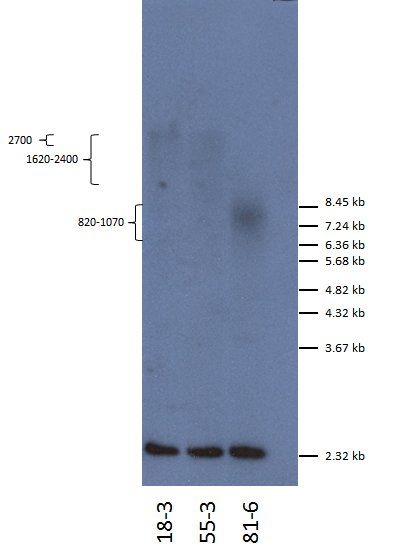

Supplement: S4 Fig — (TIF) [file pone.0162592.s004.tif]

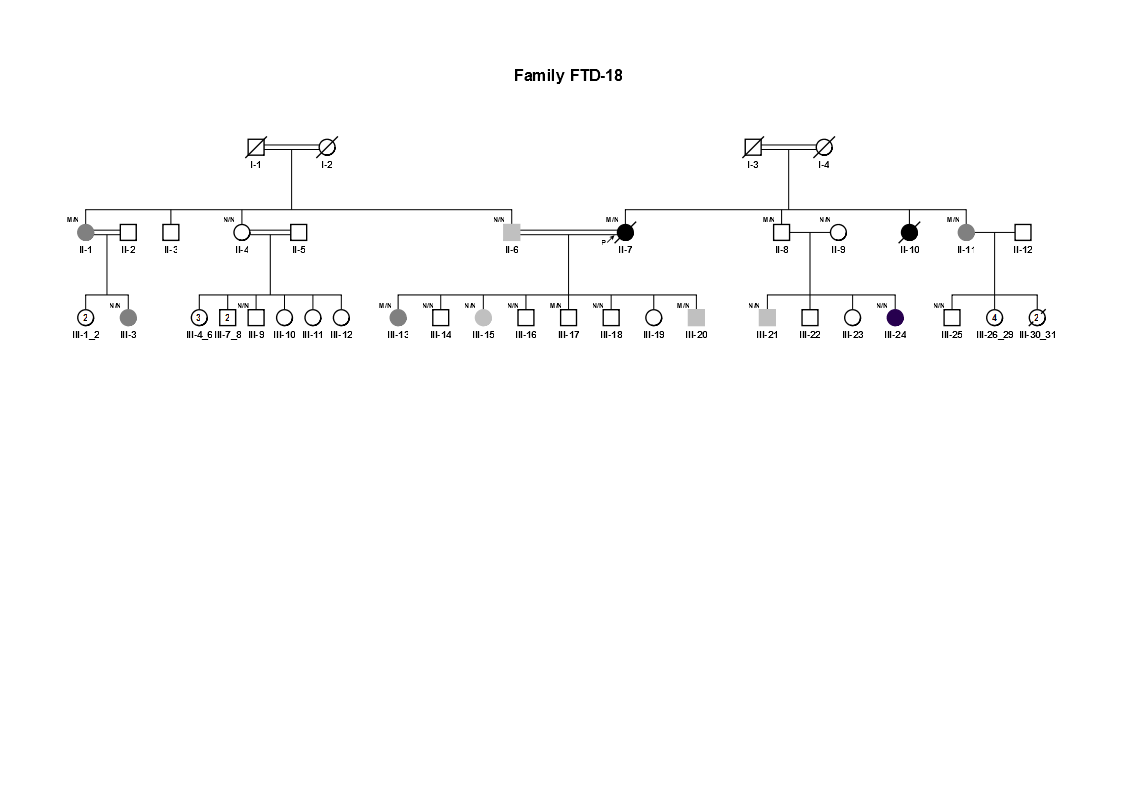

Supplement: S5 Fig — The arrow identifies the proband; dark grey symbol: major depression; light grey symbol: obessive-compulsive disorder; dark blue: mental retardation; M: C9ORF72 expansion carrier; N: wild type. (TIFF) [file pone.0162592.s005.tiff]
