# Supplementary material for: Mutation Frequency of the Major Frontotemporal Dementia Genes, MAPT, GRN and C9ORF72 in a Turkish Cohort of Dementia Patients
Source: PLoS One. 2016 Sep 15;11(9):e0162592. doi: 10.1371/journal.pone.0162592 (PMC5025192; doi:10.1371/journal.pone.0162592)
Supplement: S1 Table — *cut-off score: 86 for age < 60 years and education > 12 years. (DOCX) [file pone.0162592.s006.docx]

**S1 Table. Clinical phenotype of the assessed family FTD18 members**

| **Fam Nr** | **C9ORF72 expansion** | **Age (y)** | **Education (y)** | | **ACE-R/100*** | **SCID: Structured Clinical Interview for DSM-IV and clinical findings** |
| --- | --- | --- | --- | --- | --- | --- |
| II-7 | pathologic | 68 | | 5 | Not possible | FTD, does not speak, bedridden |
| II-11 | pathologic | 51 | | 12 | 68 | Major depression, specific phobia |
| II-8 | pathologic | 58 | | 15 | 69 | Not evaluated |
| II-6 | 0 | 69 | | 8 | 63 | Obsessive compulsive disorder, specific phobia( hight) |
| II-4 | 0 | 69 | |  | na | Not evaluated, healthy reported |
| II-1 | pathologic | 70 | | 5 | only MMSEavailable: 16/28 | Major depression, severe anxiety disorder, low performance in cognitif examination (speaks only Kurdish) |
| III-14 | 0 | 44 | | 12 | na | Not evaluated, healthy reported |
| III-13 | pathologic | 46 | | 12 | 65 | Major Depression, specific phobia (hight) |
| III-15 | 0 | 43 | | 13 | 79 | Obssesive compulsive disorder (symetry), agoraphobia, specific and social phobia (sea), |
| III-16 | 0 | 41 | | 15 | 85 | Normal |
| III-17 | pathologic | 37 | | 12 | 79 | Cognition normal |
| III-18 | 0 | 33 | | 16 | 90 | Specific phobia (sea), cognition normal |
| III-20 | pathologic | 29 | | 13 | 87 | Obsessive compulsive disorder (counting numbers), dystimia with major depression episode, social phobia, post traumatic stress disorder (after divorce) |
| III-21 | 0 | 25 | | 16 | 90 | Obsessive Compulsive disorder (symetry, counting numbers), panic disorder, cognition normal |
| III-24 | 0 | 17 | | 5 | na | Mental retardation, agoraphobia, specific phobia, |
| III-9 | 0 | 37 | | 13 | 84 | Specific phobia( hight), cognition normal |
| III-3 | 0 | 26 | | 4 | na | Major Depression, panic disorder, agoraphobia, social phobia |
| III-25 | 0 | 17 | | 11 | 79 | Social hpobia, specific phobia (dog), cognition's performance is limit, mild bilateral hand tremor |
