# Supplementary material for: Mutation Frequency of the Major Frontotemporal Dementia Genes, MAPT, GRN and C9ORF72 in a Turkish Cohort of Dementia Patients
Source: PLoS One. 2016 Sep 15;11(9):e0162592. doi: 10.1371/journal.pone.0162592 (PMC5025192; doi:10.1371/journal.pone.0162592)
Supplement: S2 Table — na: not available; dbSNP: single nucleotide polymorphism database; ID: identification. (DOCX) [file pone.0162592.s007.docx]

**S2 Table. Variants identified in *C9ORF72*, *CHMP2B, FUS, TARDBP,* *VCP* genes and synonymous variants in *MAPT, GRN***

| **Gene** | **Transcript ID** | **Position** | **Annotation** | **cDNA change** | **Amino acid change** | **dbSNP ID** |
| --- | --- | --- | --- | --- | --- | --- |
| *C9ORF72* | ENST00000380003 | 9:27550659 | missense variant | c.1138T>G | p.F380V |  |
| *C9ORF72* | ENST00000380003 | 9:27556780 | synonymous variant | c.870C>T | p.S290S | rs10122902 |
| *C9ORF72* | ENST00000380003 | 9:27561628 | missense variant | c.620A>G | p.N207S | rs17769294 |
| *C9ORF72* | ENST00000380003 | 9:27566973 | missense variant | c.146C>G | p.T49R | rs201250307 |
| *C9ORF72* | ENST00000380003 | 9:27567145 | 5’ prime UTR variant | c.-27G>A |  | rs10757668 |
| *CHMP2B* | ENST00000263780 | 3:87276699 | synonymous variant | c.27C>T | p.T9T | rs2279720 |
| *CHMP2B* | ENST00000263780 | 3:87295049 | synonymous variant | c.312T>C | p.T104T | rs11540913 |
| *CHMP2B* | ENST00000263780 | 3:87299075 | synonymous variant | c.372A>C | p.T124T | rs1044499 |
| *CHMP2B* | ENST00000263780 | 3:87303064 | 3’ prime UTR variant | c.*106delA |  | na |
| *CHMP2B* | ENST00000263780 | 3:87303064 | 3’ prime UTR variant | c.*106dupA |  | na |
| *FUS* | ENST00000568685 | 16:31191482 | 5’ prime UTR variant | c.-54A>G |  | rs929867 |
| *FUS* | ENST00000568685 | 16:31202698 | splice region variant&intron variant | c.1545-5delT |  | na |
| *FUS* | ENST00000568685 | 16:31202698 | splice region variant&intron variant | c.1545-6_1545-5delTT |  | na |
| *FUS* | ENST00000568685 | 16:31202698 | splice region variant&intron variant | c.1545-5dupT |  | na |
| *FUS* | ENST00000568685 | 16:31202800 | 3’ prime UTR variant | c.*41G>A |  | rs80301724 |
| *FUS* | ENST00000568685 | 16:31193942 | synonymous variant | c.147C>A | p.G49G | rs741810 |
| *FUS* | ENST00000568685 | 16:31195279 | synonymous variant | c.291C>T | p.Y97Y | rs1052352 |
| *FUS* | ENST00000568685 | 16:31195604 | inframe deletion | c.430_447delGGACAGC  AGCAAAGCTAT | p.G144_149del | na |
| *FUS* | ENST00000568685 | 16:31195692 | splice donor variant&disruptive inframe deletion  &splice region variant&intron variant | c.521_523+3delGAGGTG | p.G175del | na |
| *GRN* | ENST00000053867 | 17:39783156 | synonymous SNV | c.384T>C | p.D128D | rs25646 |
| *GRN* | ENST00000053867 | 17:42427037 | synonymous SNV | c.267C>T | p.A89A | rs201699327 |
| *GRN* | ENST00000053867 | 17:42428798 | synonymous SNV | c.903G>A | p.S301S | rs63750142 |
| *GRN* | ENST00000053867 | 17:42429810 | synonymous SNV | c.1515C>T | p.A505A | rs147495221 |
| *MAPT* | ENST00000344290 | 17:41416862 | synonymous SNV | c.855C>T | p.D285D | rs63750222 |
| *MAPT* | ENST00000344290 | 17:41417096 | synonymous SNV | c.1089G>A | p.P363P | na |
| *MAPT* | ENST00000344290 | 17:41427131 | synonymous SNV | c.1512T>C | p.T504T | rs62063845 |
| *MAPT* | ENST00000446361 | 17:41429726 | synonymous SNV | c.507A>G | p.A169A | rs1052553 |
| *MAPT* | ENST00000446361 | 17:41420268 | synonymous SNV | c.225C>T | p.D75D | na |
| *MAPT* | ENST00000446361 | 17:41424761 | synonymous SNV | c.354G>A | p.P118P | rs1052551 |
| *MAPT* | ENST00000446361 | 17:41429855 | synonymous SNV | c.636G>A | p.P212P | rs11568305 |
| *MAPT* | ENST00000446361 | 17:41429810 | synonymous SNV | c.591T>C | p.N197N | rs17652121 |
| *MAPT* | ENST00000334239 | 17:44101513 | synonymous SNV | c.1071C>T | p.A345A | na |
| *TARDBP* | ENST00000240185 | 1:11073982 | synonymous variant | c.198T>C | p.A66A | rs61730366 |
| *TARDBP* | ENST00000240185 | 1:11080611 | synonymous variant | c.669C>G | p.P223P | rs149517613 |
| *TARDBP* | ENST00000240185 | 1:11080611 | sequence feature | c.669C>G |  | rs149517613 |
| *VCP* | ENST00000358901 | 9:35057110 | 3’ prime UTR variant | c.*4G>T |  | rs201091341 |
| *VCP* | ENST00000358901 | 9:35060302 | splice region variant&intron variant | c.1695+8A>G |  | rs684562 |
| *VCP* | ENST00000358901 | 9:35060955 | sequence feature | c.1360-35A>G |  | rs2258240 |
| *VCP* | ENST00000358901 | 9:35061490 | sequence feature | c.1194+84G>T |  | rs74763589 |
| *VCP* | ENST00000358901 | 9:35061503 | sequence feature | c.1194+71A>G |  | rs2074549 |
| *VCP* | ENST00000358901 | 9:35061693 | splice region variant&intron variant | c.1082-18_1082-8dup  TTGTGTACTGT |  | rs11272867 |
| *VCP* | ENST00000358901 | 9:35062972 | splice region variant&intron variant | c.811+3G>A | | rs514492 |
| *VCP* | ENST00000358901 | 9:35064324 | sequence feature | c.577-42A>G |  | rs200165441 |
| *VCP* | ENST00000358901 | 9:35068201 | sequence feature | c.129+47G>A |  | rs10972300 |
